# Multi-class classification of plant leaf diseases using a hybrid deep neural transformer system and explainable AI techniques

**DOI:** 10.1038/s41598-026-48103-3

**Published:** 2026-04-19

**Authors:** Saravanan Srinivasan, Ranjith Kumar A, Nagashree B. A, Jitender Tanwar, Virendra Pal Singh, Usha Moorthy

**Affiliations:** 1 Department of Computer Science and Engineering, SRM Institute of Science and Technology, Ramapuram, Chennai India; 2 Department of Computer Applications, Vidyavardhaka College of Engineering, Mysuru, India; 3 School of Computing Science and Engineering, Galgotias University, Greater Noida, 203201 India; 4 Manipal Institute of Technology Bengaluru, Manipal Academy of Higher Education, Manipal, India

**Keywords:** Plant leaf disease classification, Cross-domain evaluation, DL, Explainable AI, Plant Leaf Disease and Transformers, Computational biology and bioinformatics, Engineering, Mathematics and computing, Plant sciences

## Abstract

An effective framework based on deep learning (DL) is developed in this study for reliable and accurate performance. The multi-class detection of crops such as corn, tomato and potato is accurate and reliable. The aim is to improve early disease detection which guarantees good classification accuracy, strong generalization across datasets, enhanced interpretability through XAI methods and enabling realistic agricultural applications. : The study uses two large publicly available datasets of plant leaf disease images of corn, tomato and potato. The first dataset designated as D-I consists of 39,203 images belonging to 18 classes of disease and the second dataset designated as D-II consists of 65,565 images also belonging to 18 classes of disease. The two datasets contain images showing different visual scenarios and variations of disease which will help form a multi-class classifier. In this study, five DL architectures were used; InceptionNetV3, ResNet152V2, ViT, BERT, and the proposed hybrid model (ResViT-152) that combined convolutional feature extraction with transformer-based global attention. Every model was trained, validated, and tested under the same experimental setup. Cross validation and multi-phase testing assessed their performances in their capacity to learn discriminative parameters in corn, tomato and potato disease classes. The hybrid model exhibited a better performance in all test conditions. In IntraTest1, the accuracies were 99.12%, 98.94% and 99.06% for corn, tomato and potato respectively. In IntraTest2, the model achieves accuracy of 99.23% for corn, 98.97% for tomato, and 98.98% for potato on D-II. The precise percentages for the cross-tests were 96.27% (corn), 95.14% (tomato), 95.06% (potato) for CrossTest1 and 95.77% (corn), 96.22% (tomato), 96.15% (potato) for CrossTest2. The performance across datasets for all three crops is good and generalization is robust. A study is conducted on an efficient, high-performing, and interpretable DL framework for plant leaf disease detection. The experimental results verify that the proposed work provides superior performance. Generalization outperforms standard architectures in effectiveness. In addition, there is also stable performance with varying datasets. With Explainability included, the model becomes more transparent and a strong candidate for further validation toward deployment in precision agriculture, pending evaluation on real-world field datasets.

## Introduction

Agriculture is one of the important components responsible for the production of food. Crop health is considered essential for better production and long-term sustainability^1^. The plant disease detection is important because it will help to avoid significant damage to the crop and reduction in production^2^. Many farmers still check their crop fields manually, which takes a lot of time and effort. Besides, the outcome is not always uniform. The process is further complicated by differences in disease expression, weather patterns and pests^3^. As the practices of farming keep changing, the requirement of smart and automated systems for the farmers keep developing^4^. By using modern computation, the cost and time involved in disease diagnosis can be minimized. By using these techniques there is a bridge formed between traditional farming limitations and modern-day agriculture^5^. Corn, tomato and potato are amongst the most cultivated crops in the world. Together, they contribute significantly to the food supply chains as well as agricultural economies^6^. Crops are susceptible to several diseases affecting their leaves, stems and other parts. Tomato leaves have early blight, late blight, and virus diseases, whereas corn is commonly infected by rust, leaf blight, and spot diseases^7^. In a similar vein, potato plants have been under serious threat from several diseases. Farmers cannot consistently and reliably identify diseases due to the large variation of symptoms^8^. Through accurate and immediate classification, this automated disease detection may lessen these issues. Various systems can be used to intervene early and increase production^9^. There are various symptoms that often appear which include discoloration, local lesions, streaks, blight, irregular shapes, and mosaic. According to the type of crop, type and stage of disease, these symptoms can vary considerably^10^. Humidity, temperature, soil quality and light conditions change the visual features of leaves. Classes share visually similar characteristics because of these variations and introducing additional sub-class variations^11^.

Using manual observations to interpret signs, especially if subtle, can easily lead to false assessments. Thus, we need cameras which can accurately interpret these events. Crop management is determined and illness identification is aided by these systems^12^. Significant progress in DL has aided in plant disease detection through images. CNNs can locate the local features such as texture, edges and speck patterns from the images of leaves^13^. Transformer-based models, on the other hand, are adept at learning global relations, allowing them to capture leaf-wide contexts^14^. The use of these techniques enables to capture local anomalies and global structures changes. By improving its robustness, the model can better adapt to different datasets and diseases^15^. DL frameworks are designed to scale up and work with large datasets. Real-time detection of diseases in precision agriculture has been supported with superior and effective solutions for this^16^.

Existing approaches show several serious limitations despite significant advances. A majority of CNN-based models are merely benchmarked on controlled datasets like PlantVillage and are not generalizable to field conditions. While Pure Transformer architecture possesses strong global reasoning capabilities, it struggles with extracting fine-grained local features, with large datasets being provided during training. There are few hybrid approaches, and existing works lack rigorous cross-dataset evaluation^28,29^. Most of the methods have no interpretability mechanism making it difficult for agricultural applications. These gaps motivate the proposed ResViT-152: a hybrid CNN–Transformer model evaluated across two heterogeneous datasets using four test protocols, with integrated Grad-CAM + + explainability.

### Problem statement

Diseases of crop leaves are one of the serious threats to a few essential crops such as corn, tomato and potato. This is due to the reasons that the disease can spread fast and unnoticed at early stages of the disease cycle. This significantly reduces food production globally. The routine manual inspection methods are slow, subjective, and inconsistent particularly due to disease symptoms of multiple classes being visually similar. A more accurate diagnosis for the leaf disease is complicated due to the high variability of the leaves, environmental conditions, and the quality of the image. Currently, automated systems struggle to generalize, particularly when datasets differ in scale, lighting, and disease distribution. Farmers or agronomists determine that unreliable automated predictions are not trustworthy due to a lack of high accuracy and interpretability models. As such, there is a requirement for a robust, generalizable, and explainable DL system that can classify plant leaf disease successfully across distinct dataset and crop categories.

### Study novelty

The proposed study makes five key contributions. First, ResViT-152 fuses ResNet152V2 and a ViT-style encoder to jointly capture local lesion textures and global leaf-level context a combination not previously explored for multi-crop disease classification. Second, the model is evaluated through four protocols (IntraTest1, IntraTest2, CrossTest1, CrossTest2) across two large datasets (D-I: 39,203 images; D-II: 65,565 images), explicitly testing cross-dataset generalization absent in prior works. Third, a unified framework simultaneously classifies 18 disease classes across corn, tomato, and potato under a consistent pipeline. Fourth, Grad-CAM + + provides spatially precise disease region visualization supporting expert interpretability. Fifth, CutMix, MixUp, and AdamW with weight decay jointly address class imbalance and overfitting persistent challenges in agricultural image datasets. Unlike recent hybrid architectures such as EfficientNetV2 + Transformer and DSKN^29^, which lack cross-dataset validation, omit interpretability, or address single-crop classification only, ResViT-152 uniquely integrates CBAM attention, Grad-CAM + + explainability, and four-protocol cross-dataset evaluation across three crops simultaneously.

## Related work

Rabbia Mahum et al.^17^ automated potato disease classification system to reduce crop loss and lessen manual assessment. Earlier productions deal with using images from PlantVillage and DL (DL) models, like improved DenseNet versions, to detect potato diseases with improved efficacy, very often just using reweighted losses to better deal with class imbalance.

Mohit Agarwal et al.^18^ tomato diseases have received considerable attention due to their impact on production. In earlier studies, lightweight CNN architectures were designed and classic machine learning algorithms like k-NN, Decision Trees were applied on PlantVillage with 10 classes of tomato diseases. Convolutional neural network (CNN) accuracy is around 98.4% which is better than heavier approach with pre-trained network with VGG16. In comparison to traditional machine learning methods that after a moderate level of accuracy apply.

Oppenheim D et al.^19^ visual symptoms enable DL–based models to classify plant diseases efficiently. The first experiments on detecting potato diseases used tubers from cameras (RGB low-cost sensors) manually assembled datasets and trained deep CNNs to distinguish a range of diseases and healthy samples. These models were extremely accurate in all the train–test splits, establishing the feasibility of deep convolutional networks tuber-level disease identification.

Rajasekaran Thangaraj et al.^20^ the process of spotting tomato leaf disease has largely depended on experts look-eyeing up close. The first automated techniques used were classical image processing and machine-learning techniques. As a result, such methods have limited accuracy due to shallow extraction of features. Recent surveys demonstrate a shift towards DL models which offer significantly high-performance recognition for tomato diseases with the aid of large public and private tomato disease datasets and CNN/Transformer based architectures.

Ruchi Gajjar et al.^21^ systems for crop disease detection in real-time using DL and embedded hardware platforms. The previous work consists of CNN based classifiers working in conjunction with single shot detectors to identify disease and localize the leaf simultaneously. It was installed on the NVIDIA Jetson TX1 for deployment. After field trials, these systems boast a 96.9% accuracy in classifying a disease, showing the practicality of these systems.

Amal Jlassi et al.^22^ Strategies like MobileNetV2 seek to improve early disease detection on potato leaves, which achieved a good performance on benchmarks such as PlantVillage. By fine-tuning lightweight pretrained architectures combined with data augmentation, reweighting strategies, and XAI-based interpretability, these methods deal with class imbalance and overfitting. The classification accuracy of the reported results is found to be greater than 98% with the same Recall and precision across diseases.

Sk Mahmudul Hassan et al.^23^ Hybrid DL and ensemble models are used to classify plant diseases in corn potato tomato crops. The study shows a shallow VGG architecture with XGBoost gives better results than hand-crafted feature methods and deep CNN models. Corn received an accuracy of 94.47 per cent, potato 98.74 per cent and tomato 93.91 per cent. On the actual scene images, the model achieved average accuracies of 94.22%, 97.36% and 93.14% with a good performance.

Wubetu Barud Demilie et al.^24^ plant disease detection has shifted from traditional manual inspection to an automated system based on machine learning and DL algorithms. Classical ML techniques like SVM, KNN, and ANN have shown moderate success to achieve the desired results, whereas it has been observed that the DL models CNN-based can extract spatial features and achieve higher classification performance. Although we have made quite a few advancements, the dataset generalization of many techniques is still weak.

Zhiwen Tang et al.^25^ Due to the differences between classes and their similarity, tomato leaf diseases can be difficult to detect. LPNet, an advanced object-detection framework, adopts perceptual adaptive convolutions. Attention modules and nearby feature aggregation improve the signal-to-noise ratio in the framework. The PLPNet model was tested on a self-constructed dataset, obtaining a mAP@50 of 94.5%, recall of 54.4%, and speed of processing of 25.45 FPS. According to the results obtained, it is evident that the proposed technique proved better than many well-known object detection models on the same dataset.

Jadhav S et al.^26^ the limitations of traditional machine learning approaches for plant leaf disease detection due to inadequate feature representation. To address this issue, a cascaded deep convolutional neural network (CDCNN) was proposed to enhance feature discrimination by reducing intra-class variability and increasing inter-class separability. The model demonstrated strong performance on the PlantVillage dataset across multiple crop diseases and further achieved high accuracy when evaluated on real-time cotton leaf disease datasets, outperforming both conventional machine learning and standard DL models.

Pantazi X et al.^27^ a technique for automatic detection of crop infections was introduced which makes use of the Local Binary Pattern features and one-class classification models using separate classifiers for each crop disease. The deliberate process exhibited high cross-crop generalization assisted by a conflict resolution for ambiguous cases, achieved an overall success of 95% over 46 plant–condition combinations.

Krishna M S et al.^28^ to overcome the generalization problems present in real-world scenarios, a framework for detecting plant diseases was created from PlantDoc dataset and images obtained from web source. Improved cross-dataset performance was delivered by fine-tuned CNN architectures, such as EfficientNet and ResNet variants, and enhanced data augmentation strategies. The results confirmed that augmenting and diversifying datasets increases the robustness of a model in uncontrolled conditions.

Kulkarni N P et al.^29^ a multi-class plant leaf disease detection using a Deep Spiking Kronecker Network (DSKN) is presented. The method included preprocessing images, segmentation, augmentation, feature extraction. However, disease classification took place at the base station. Results of experiments on PlantVillage and crop disease image datasets showed competitive performance with an accuracy of 90.76% with good true and false positive rates.

Kalpana et al.^32^ proposed an ensemble heterogeneous Transformer model for multi-class plant disease diagnosis, demonstrating that combining diverse Transformer architectures improves classification robustness.

Kalpana and Anandan^33^ introduced a capsule attention network for plant disease classification, showing that attention-based feature refinement effectively discriminates between visually similar disease classes. While these studies confirm the value of attention and Transformer mechanisms, they do not incorporate cross-dataset evaluation or integrated XAI explainability gaps directly addressed by the proposed ResViT-152.

Kalpana et al.^34^ applied SE-ResNet152 with transfer learning for early corn leaf disease identification, achieving competitive classification performance. However, the study focused exclusively on corn under a single-dataset setting, without extending to multi-crop or cross-dataset evaluation.

Srinivasan et al.^3^ proposed DBA-ViNet for fruit disease detection using explainable AI, demonstrating the effectiveness of DL frameworks with XAI visualization in agricultural applications. Murugesan et al.^9^ presented a hybrid DL approach with Grad-CAM interpretability for multiclass crop leaf disease classification, reporting competitive performance across multiple crop categories. Baiju et al.^5^ demonstrated robust leaf disease detection for CRW crops using hybrid DL models. While these recent studies confirm the growing adoption of hybrid and explainable approaches, they focus on single-dataset evaluation and do not address simultaneous multi-crop classification with rigorous cross-dataset generalization testing and gaps that the proposed ResViT-152 directly targets Table 1.

**Table 1:** Summary of existing DL approaches for plant disease classification.

Author	Model	Purpose	Observation
^17^	Improved DenseNet variants with reweighted loss	Automated potato disease classification to reduce crop loss	Evaluated mainly on Pla+E4:E14ntVillage; relies on class reweighting and lacks cross-dataset generalization analysis
^18^	Lightweight CNN, k-NN, Decision Tree, VGG16	Tomato disease classification using PlantVillage dataset	Traditional ML shows moderate accuracy; CNN performs well but limited to controlled dataset conditions
^19^	Deep CNNs on RGB tuber images	Potato disease identification at tuber level	Uses manually assembled datasets; high accuracy but limited validation on diverse field conditions
^20^	CNN- and Transformer-based DL models	Automated tomato leaf disease detection	Survey highlights performance gains, but earlier shallow models suffer from weak feature representation
^21^	CNN + Single Shot Detector on Jetson TX1	Real-time crop disease detection and localization	Achieves good accuracy but constrained by embedded hardware resources and scalability
^22^	MobileNetV2 with data augmentation and XAI	Early potato leaf disease detection	Performance evaluated on PlantVillage; limited testing on real-field images
^23^	Hybrid VGG + XGBoost ensemble	Multi-crop disease classification (corn, potato, tomato)	Strong performance but accuracy drops on real field images, indicating generalization issues
^24^	Classical ML (SVM, KNN, ANN) and CNN	Automated plant disease detection	Classical ML shows limited performance; DL improves accuracy but generalization remains weak
^25^	LPNet with attention and adaptive convolutions	Tomato disease detection using object detection	Focuses on detection rather than classification; evaluated on self-built dataset only
^26^	Cascaded Deep Convolutional Neural Network (CDCNN)	Multi-class plant leaf disease classification using deep feature learning	High accuracy is achieved on curated datasets; however, robustness under cross-dataset or real-field conditions is not extensively validated.
^27^	Local Binary Pattern (LBP) + One-Class Classifiers	Automated crop disease identification with cross-crop generalization	Demonstrates strong generalization and effective conflict resolution; reliance on handcrafted features limits scalability to complex disease patterns.
^28^	Fine-tuned CNN models (EfficientNet, ResNet variants)	Plant disease detection under uncontrolled, real-world conditions	Dataset diversity and augmentation improve generalization; overall accuracy remains moderate compared to recent hybrid architectures.
^29^	Deep Spiking Kronecker Network (DSKN) with IoT	Multi-class leaf disease detection in IoT-based agriculture	Competitive performance is reported; system complexity and limited interpretability restrict practical deployment.

The reviewed literature reveals four persistent gaps that the proposed study directly addresses. First, most methods^17,18,22,26^ are evaluated on single controlled datasets, lacking cross-dataset generalization validation. Second, pure CNN architectures^18,19,23^ fail to capture global leaf-level context, while pure Transformer models^20^ struggle with fine-grained local lesion features. Third, interpretability through XAI is either absent or limited to basic Grad-CAM without disease-specific spatial validation^9,22^. Fourth, no existing study simultaneously addresses multi-crop classification across corn, tomato, and potato with dual-dataset cross-evaluation and integrated explainability within a unified framework. ResViT-152 is specifically designed to close all four of these gaps.

## Materials

The dataset applied for the procedure consists of large image data of corn, potato, and tomato leaves that belong to various popularly known disease classes. Two datasets are important: D-I^30^ and D-II^31^. For each class, a stratified image-wise partitioning of 70% training, 15% validation, and 15% testing was applied. Since both D-I and D-II are publicly available Kaggle datasets consisting of independently captured leaf images not sequential frames from the same plant each image represents a unique sample, ensuring no plant-level overlap between splits. Augmentations were applied exclusively within the training set after splitting, eliminating any risk of data leakage between training and test subsets. The disease categories of CCLSL, CCR, CH, and CNLB has 1436–1669, and 1845–2587 training samples for corn of D-I and D-II respectively as shown in Table 2. The validation and test samples shown proportional. The D-I potato classes PEB, PH and PLB fetched 1,596-1,696 training images while D-II fetched 1,712-2, 475 images. Tomato is registered having larger disease types such as TBS, TEB, TH, TLB, TLM, TSLS, TSMTSM, TTS, TTMV and TTYLCV and their respective training samples are D-I 1489–1715 and D-II 1868–5522. The systematic spread of diseases in both the datasets ensures an adequate representation of their variations which will provide a good base for training, validating and generalizing the model across all selected crops. D-I and D-II were specifically selected because they represent two distinct visual distributions of the same disease classes enabling cross-dataset generalization testing not possible with a single benchmark. D-I offers highly curated and consistent image conditions while D-II introduces variation in lighting, quality of camera, the stage of disease progression to simulate diversity seen in agricultural applications in the real world. Both datasets include RGB JPEG images were retrieved from Kaggle. D-I includes laboratory photos with certain lighting and uniform backgrounds, whereas D-II contains field-acquired samples with variable illumination, cluttered backgrounds and mixed camera resolutions conditions closely reflecting typical agricultural settings. The difference in how the data are acquired makes for a difficult and realistic dual-dataset evaluation that tests cross-dataset generalization, ensuring the proposed model is verified.

**Table 2:** Summary of image distribution of datasets.

Leaf	Class	Train (70%)		Valid (15%)		Test (15%)	
		D-I	D-II	D-I	D-II	D-I	D-II
Corn	CCLSL	1436	2587	308	554	308	554
	CCR	1668	1845	358	395	358	395
	CH	1626	2442	349	523	349	523
	CNLB	1669	2493	358	534	358	534
Potato	PEB	1696	2475	364	530	364	530
	PH	1596	1712	342	367	342	367
	PLB	1696	2472	364	530	364	530
Tomato	TBS	1489	3056	319	655	319	655
	TEB	1680	2445	360	524	360	524
	TH	1685	2846	361	610	361	610
	TLB	1620	3035	347	650	347	650
	TLM	1646	2378	353	510	353	510
	TSLS	1527	1868	327	400	327	400
	TSMTSM	1524	3439	326	737	326	737
	TTS	1598	2699	343	578	343	578
	TTMV	1566	2583	336	554	336	554
	TTYLCV	1715	5522	368	1183	368	1183

**Fig. 1:**
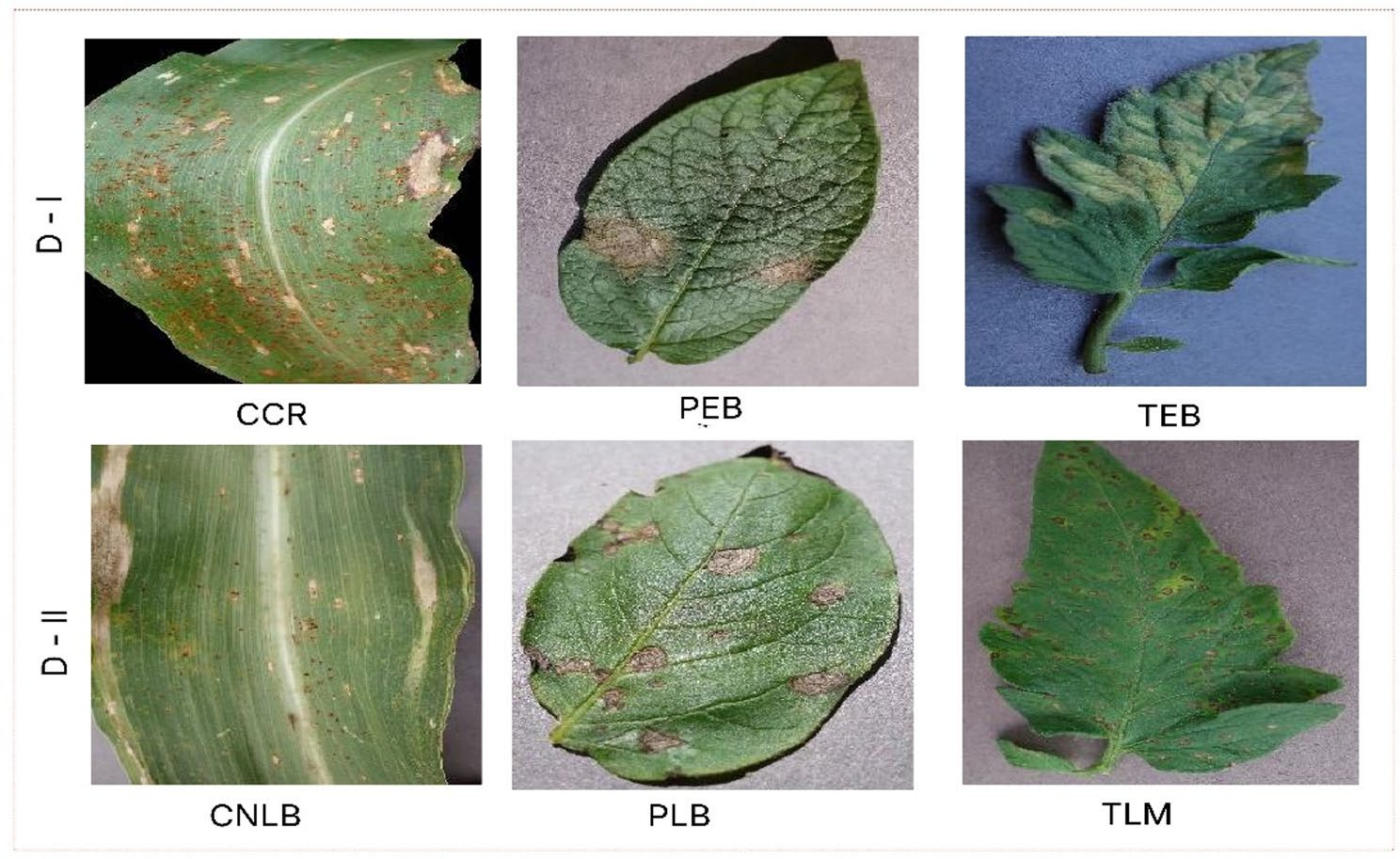
Images of two datasets with respective classes.

While the datasets are quite diverse in terms of the diversity of leaf disease class, yet difference between D-I and D-II open a possibility of image bias due to lighting, background condition, camera quality, disease appearance etc. Some classes of tomato in D-II contains much larger number of images than others. It can lead to overfitting during training because of the class imbalance effect. To overcome these challenges, we applied four strategies: (1) stratified 70/15/15 splitting to maintain class proportions; (2) label smoothing (ε = 0.1) to avoid over-confidence to the majority classes; (3) CutMix and MixUp augmentation to increase minority class representation on training; and (4) cross-dataset testing as shown in Fig. 1 to confirm learned decision boundaries are not data set dependent.

### Data acquisition and preprocessing techniques

To achieve a specific goal, the model has to learn various discriminative features of plant leaf images. Preprocessing the training and testing images makes it all possible. There are various strong Pre-processing techniques incorporated in this study for intensification of efficiency images in the D-I and D-II. The ResNet152V2 backbone and ViT patch embedding layer were used on all images that were resized to 224 × 224. The pixel values were normalized as per ImageNet statistics (mean = [0.485, 0.456, 0.406]; std = [0.229, 0.224, 0.225]) to stabilize convergence with pre-trained CNN weights. To lessen fluctuations of natural light from optical acquisition in field samples brightness correction and contrast normalization were performed. We reduce the model’s consideration of features lying outside of leaf and soil. Moreover, they are more significant towards disease-causing. We chose to sharpen and enhance the colors of the images to display the subtle discoloration and texture irregularities associated with disease expression. To increase robustness, the following augmentation parameters were applied during training: random horizontal and vertical flips (*p* = 0.5), random rotation (± 30°), brightness and contrast jitter (factor ± 0.2), Gaussian noise (σ = 0.01), CutMix (α = 1.0), and MixUp (α = 0.2). All augmentations were applied online per batch within the TensorFlow/Keras pipeline, ensuring no leakage between training and validation splits.

**Figure:**
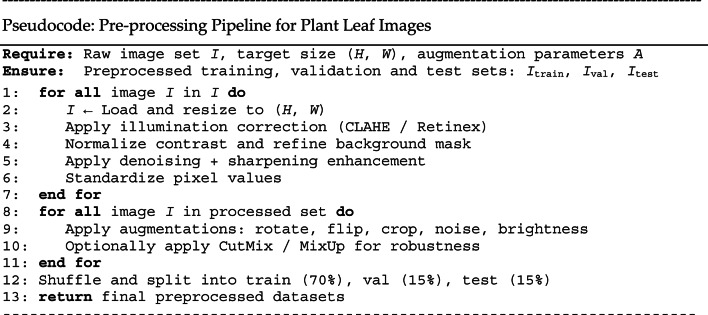
.

## Proposed hybrid model

A hybrid model is necessary to overcome the respective shortcomings of CNN’s and Transformer’s architectures. CNNs are good at capturing details like textures and fine spatial features, but no longer-range dependencies across the image. Unlike CNNs, Transformers model the global context well, but they fail to model low-level features, such as edges, veining and lesion texture. Due to the mixture of local symptoms and global phenotypic patterning, it is not sufficient to use single-architecture systems for plant leaf disease modelling. A blend of both will capture both types of information, enhancing overall representational strength. ResNet152V2 and ViT were selected due to their complementary strengths.

The ResNet152V2 architecture employs residual learning for effective feature extraction from pathological images. It exhibits a high level of stability during training and performs well on lightweight pattern recognition tasks. The ViT, in contrast to CNN, uses a multi-head self-attention mechanism that supposedly enables it to comprehend global relation across space. Thus, the model can understand long-distance relationships and disease spread over a larger leaf surface area. These models may work together in addressing local visual aspects of disease as well as global aspects.

To enable the model to learn all the complex plant disease symptoms, it is important to integrate both CNN and Transformer. The CNN layers are involved in capturing spatially localized symptoms like small lesions, texture patterns or discoloration. Similarly, the transformer layers analyze structural changes on the global symmetry level through spatial correlations of parts of the leaf. Merging the results of the two models helps the hybrid model obtain both spatial detail and holistic context. The combined approach makes feature space more balanced, which differentiates more well between visually-similar disease classes, reducing likely misclassification.

**Fig. 2:**
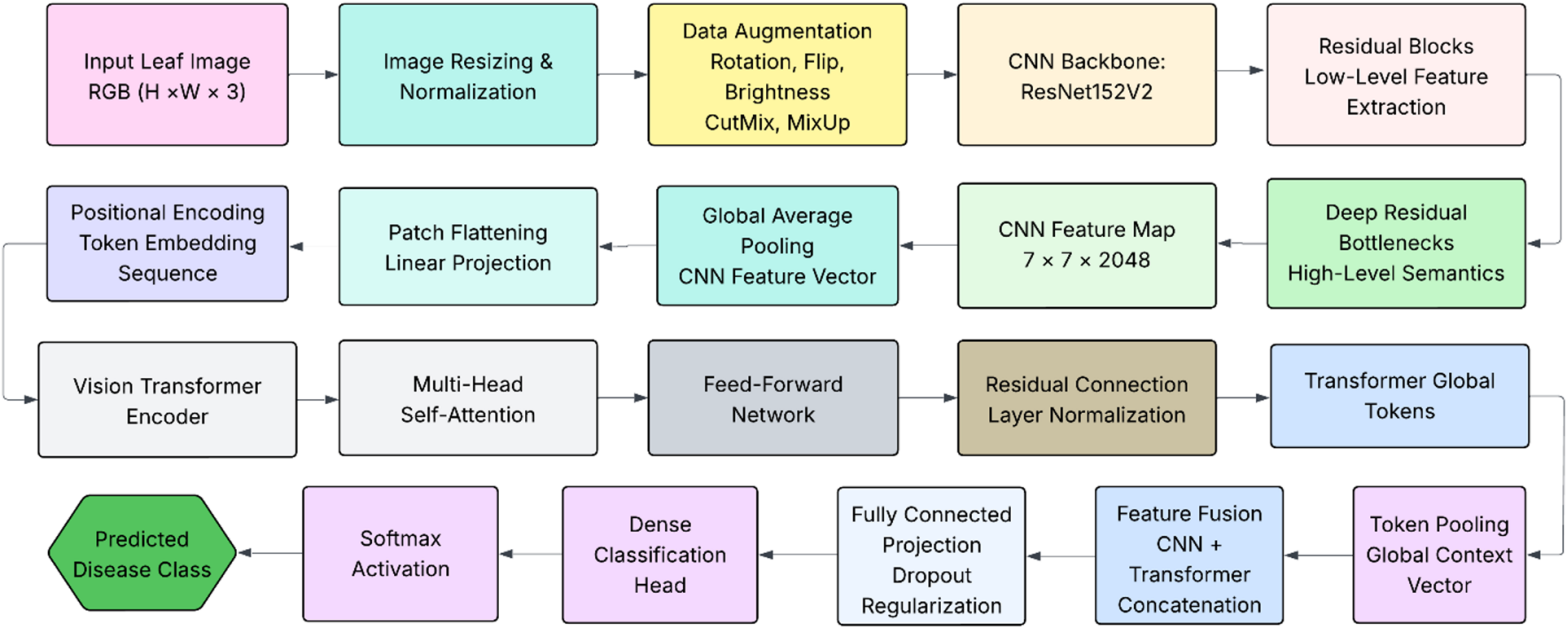
Overall architecture of proposed study.

The new fusion creates unified feature embeddings from CNN local features and transformer global contexts, as illustrated by Fig. 2. As a result, the model gets more robust and can identify disease signs in more complex scenarios. The selected design incorporates principles that have been shown to work in others. In addition, multiple models build a rich multi-scale feature hierarchy that cannot be achieved by a single model. Similarly, dissecting typical concealed traits through residual links, position encoding, and multi-headed focus. These newly generated features from spatial fusion are unique in nature. Likewise, the disease classes are more distinct (more linear) than with other approaches. Consequently, there is an enhancement in classification results.

The proposed hybrid design is a very powerful design for deep convolutional encoding and transformer-based attention processes for overall feature extraction. It effectively captures details of fine-texture diseases, large patterns, and global context. The initialization of parameters in a pre-trained manner and fusing the layers prevents any drastic fluctuations in learning and accelerates overall loss convergence. Additionally, using multiple types of residual blocks and attention heads together enhances the generalization ability of the model on natural images from a variety of visual domains. As a result, the model achieves greater accuracy, less overfitting, and better cross-domain performance when compared to using CNN or Transformer models on their own, which makes it suitable for real-world plant disease detection.

The ResNet152V2 backbone flattens the 2048 channel 7 × 7 spatial feature map and linearly projects it into 768-dimensional patch tokens (includes learnable positional encodings). The tokens are processed in six MHSA blocks (with 8 heads and GELU activation) to obtain the global embedding of 768 dimensions. At the same time, the CNN output is globally averaged to a 2048-dimensional local feature vector. Layer Normalization is applied independently to both representations and are concatenated into 2816 dimensions. A fully connected layer followed by Dropout (0.2) project this to a unified embedding of 512 dimensions. This design encodes local lesion textures and global structural patterns jointly into a single discriminative representation for the classification head.

Prior to fusion, a Convolutional Block Attention Module (CBAM) is applied to the ResNet152V2 feature maps. Channel-wise attention recalibrates responses by emphasizing disease-relevant feature channels, while spatial attention suppresses background and non-lesion regions. This dual-stage refinement ensures only diagnostically relevant features enter the fusion layer. As confirmed by ablation setting A5 (Table 5), removing CBAM reduces IntraTest accuracy by 0.72% and CrossTest accuracy by 1.41%.

**Table 3:** Technical parameter details of proposed model.

Component	Architecture	Key Parameters	Output
CNN Backbone	ResNet152V2 residual + bottleneck layers	~ 60 M params, ReLU + BN, He-normal init	Extracts local spatial features
Feature Map	7 × 7 final CNN feature grid	2048 channels	Input for patch embedding
Transformer Encoder	ViT-style MHSA + FFN + LayerNorm	6 blocks, 8 heads, GELU	Produces global contextual features
Patch Embedding	Linear projection + positional encoding	Patch dim: 768	Converts CNN map into token sequence
Feed-Forward Network	2-layer MLP (768→3072→768)	~ 20 M params	Enhances semantic representation
Fusion Layer	Hybrid concatenation (CNN + ViT)	Linear proj: 1024→512, Dropout 0.2	Creates unified hybrid embedding
Classification Head	GAP + Dense + Softmax	~ 12 M params, Xavier init	18-class output
Regularization	Dropout + Label smoothing + Early stopping	Dropout 0.2, LS 0.1	Prevents overfitting
Optimizer	AdamW + CutMix + MixUp	Weight decay 0.01	Stabilizes large model training
Overall Size	-	≈ 92 M total parameters	-

Table 3 summarizes the architectural and training parameters of the proposed ResViT-152 hybrid model. The CNN backbone (ResNet152V2) produces high-dimensional spatial features. These are reshaped into a 7 × 7 feature grid and transformed into tokens compatible with the latter. Through a linear projection layer, this takes place. These tokens go through an encoder in a ViT style consisting of six self-attention blocks to capture global context. The outputs from the CNN and the Transformer are concatenated through a fusion layer which projects them into a unified hybrid representation of dimension 512 before classifying via a dense softmax head. Various additional components like dropout, label smoothing, AdamW optimizer and MixUp/CutMix augmentation were introduced to further enhance the model. The architecture consisting of ~ 92 million parameters is developed for multi-class plant-leaf disease detection.

**Figure:**
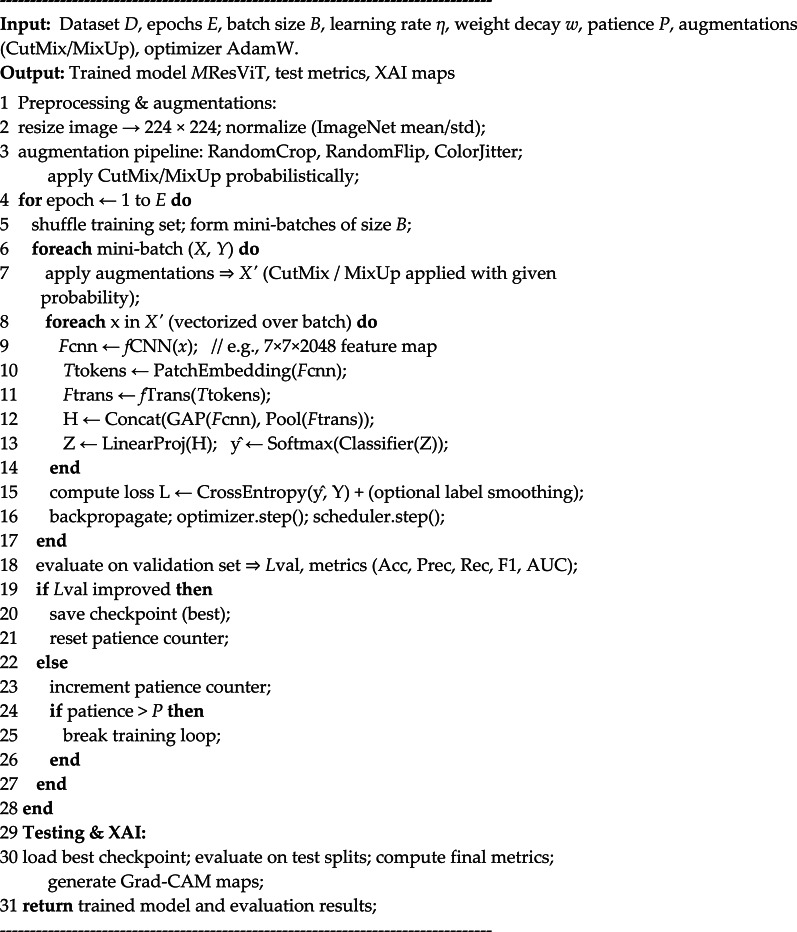
**Algorithm 1:** Training & Inference for Proposed ResViT-152.

### Algorithm 1

presents the full training and inference pipeline of ResViT-152 hybrid model. Image resizing, normalization and advanced augmentations, like CutMix, and MixUp, are applied at the start to prevent overfitting. In every epoch, the rich hybrid embedding for classification is created by extracting CNN-based local features from the ResNet152V2 backbone and global contextual features from the Transformer encoder. To gain optimal convergence without consuming unnecessary computation, we employ an early-stopping mechanism driven by validation. To make it explicit, at the end of training, the best checkpoint evaluated on the test set generates Grad-CAM maps for explainable predictions.

**Fig. 3:**
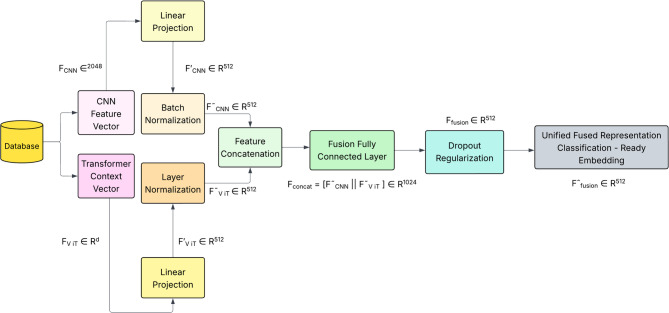
Feature-level fusion of CNN and Transformer representations.

Figure 3 illustrates the feature-level fusion strategy employed to combine CNN and Transformer representations. CNN features capturing local spatial patterns and Transformer features encoding global contextual information are first projected into a common embedding space and normalized independently. The fused features are combined to create a combined representation, which is produced through a fully connected fusion layer followed by dropout. This fusion scheme helps to learn complementary features and forms a robust embedding for classification purposes.

### Justification of proposed model over pre-trained models

ResViT-152 is different from existing CNN–Transformer hybrids in three ways. To start with, ResViT-152 applies independent Layer Norms on both the CNN and the Transformer streams prior to fusion, unlike EfficientNetV2 + Transformer hybrids that perform late concatenation. This ensures only compatible representations get fused. Secondly, the CBAM attention module refines the CNN feature maps prior to fusion and suppresses the noise coming from the background and focuses on the disease regions, such a step is not present in the hybrids based on the Swin Transformer. In addition, the diverse datasets of ResViT-152 were tested on four test protocols, measuring explicitly cross-dataset generalization, which other hybrids do not have. When combined, these distinctions yield the highest CrossTest accuracy of 96.27% (minimum stability deviation of 0.008) and highest XAI interpretability score of 93.5% as compared to all other models in Table 6.

### Experimental configuration

The training of CNN and Transformer based architecture is done on Windows 11 OS with 16 GB RAM and an NVIDIA GPU. The programming environment was developed in Python 3.10 using TensorFlow/Keras and PyTorch, supported by NumPy, Pandas, scikit-learn, and OpenCV for preprocessing, augmentation and evaluation. To ensure stable convergence, the pipeline was standardized across all models including ResViT-152. Training was conducted for 50 epochs with a batch size of 32 the maximum feasible under available GPU memory while maintaining stable gradient estimates for the Transformer attention layers and a learning rate of 1 × 10⁻⁴, which provided optimal convergence stability across both datasets as confirmed in Table 4. AdamW was selected over standard Adam for its decoupled weight decay, which prevents over-regularization of frequently updated parameters in a large (~ 92 M parameter) model. CutMix and MixUp augmentation strategies were applied to reduce dataset-specific pattern memorization, directly improving cross-dataset generalization. Early stopping monitored validation loss with a patience of 10 epochs to prevent overfitting while preserving model capacity. This configuration ensures a reliable, reproducible, and fair benchmarking environment across all evaluated architectures. Although ResViT-152 contains ~ 92 M parameters, its computational cost is justified by its performance gains. With 12.2 GFLOPs and an inference time of 10.1 ms per image, it is faster than ViT-B/16 (14.9 ms) while achieving higher accuracy and XAI interpretability. Training converged in 20 epochs with AdamW + CutMix/MixUp earlier than Adam (28 epochs) and SGD (38 epochs) as shown in Table 4 reducing overall training cost. The 4.1 GB VRAM requirement is within the range of widely available mid-range GPUs, confirming practical deployability without specialized hardware. The evaluation uses four protocols. IntraTest1 trains and tests on D-I; IntraTest2 trains and tests on D-II both measuring within-dataset performance. CrossTest1 trains on D-I and tests on D-II; CrossTest2 trains on D-II and tests on D-I both measuring bidirectional cross-dataset generalization under unseen data distributions.

## Experimental results

The proposed ResViT-152 achieves IntraTest accuracy of 99.12%, 98.94%, and 99.06% for corn, tomato, and potato respectively on D-I, with corresponding F1-scores exceeding 99.1% across all classes. Under cross-dataset evaluation, CrossTest1 yields 96.27% (corn), 95.14% (tomato), and 95.06% (potato), while CrossTest2 yields 95.77%, 96.22%, and 96.15% respectively consistently outperforming all baseline CNN and Transformer models evaluated under identical conditions. These results confirm that the hybrid fusion of local and global features produces a more discriminative and generalizable representation than either architecture alone.

**Fig. 4:**
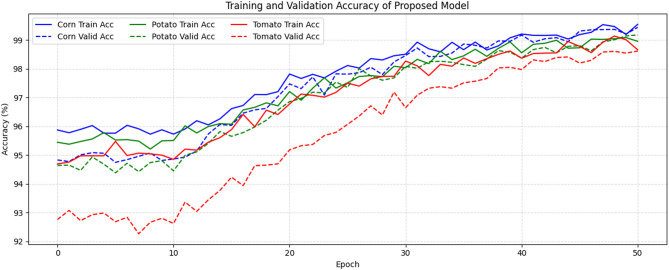
Training phase analysis of Corn, Potato, Tomato (D- I).

**Fig. 5:**
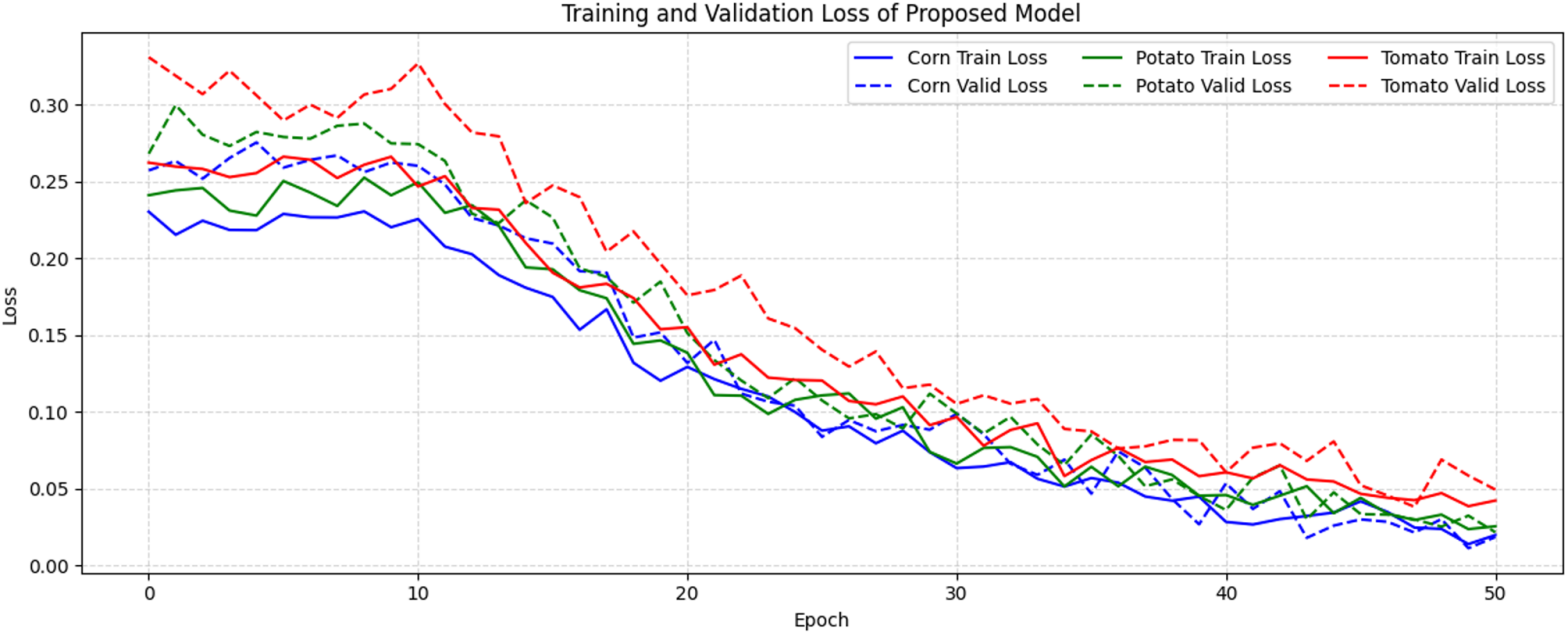
Training phase analysis of Corn, Potato, Tomato (D- II).

Figure 4 represents the analysis training phase of the model proposed for corn, potato, and tomato D-I dataset which shows the consistent pattern of performance enhancement of all the crops. As the epochs increase, both the training and validation accuracies also increase and their corresponding losses decrease, suggesting that the learning is effective and stable. The model displays a strong early fit in the first epochs and continues carving further features representation during the training process. The training and validation curves of all the three crops are smooth and highly parallel which shows the low overfitting. The corn and potato converge somewhat faster as these two crops have more distinct symptoms. Whereas, tomato has less rapid, but stable convergence across epochs. The findings show that the hybrid model produces a set of strong and discriminative features over all crop categories during the training phase Fig. 5.

It is seen in the D-II data set that the success rate of corn, potato and tomato crops increase with training. The accuracy of training and validation rises while losses continue to reduce. This demonstrates effective learning with strong convergence. The size of the dataset being larger. Results from D-II provide an indication of much slower improvement in the early epochs in comparison to the performance witnessed in D-I. D-II reflects higher complexity and variation because of the extended or longer dataset. The training and validation curves for all three crops are almost similar which indicates slight overfitting. The disease pattern in corn is a little more distinct, enabling corn to converge slightly faster than tomato. Journey through ages of corn learning. In conclusion, the results show that the proposed hybrid model would maintain stable performance and scalability when trained on larger and more diverse datasets.

**Fig. 6:**
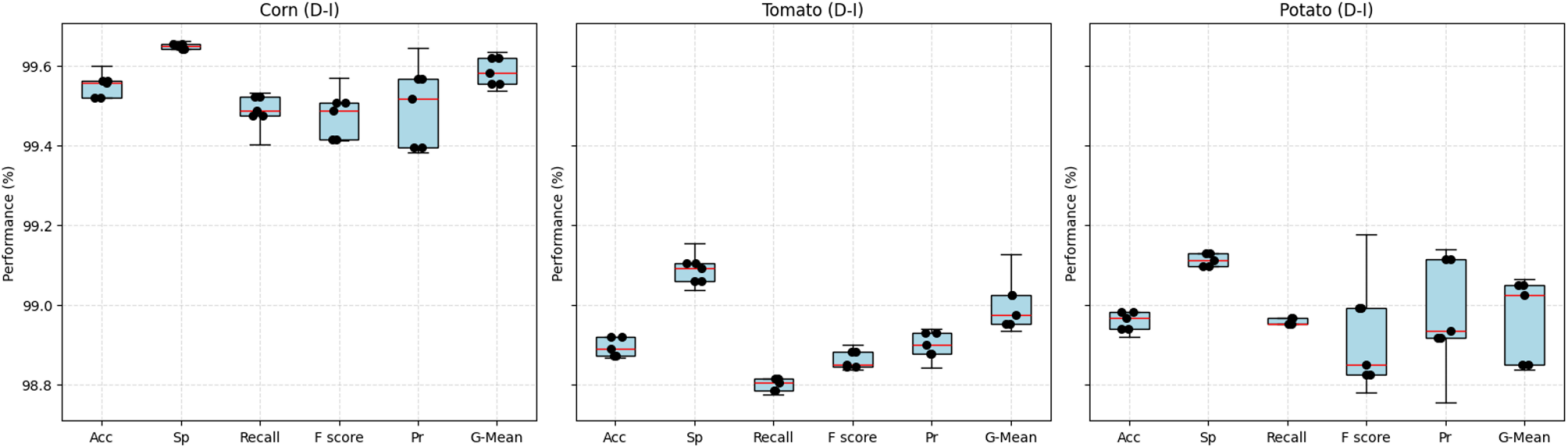
Five-fold cross-validation analysis of the proposed model on D-I.

Figure 6 presents a box plot showing performance distribution of the proposed model for five folds. Black dots represent the results of the cross-validation for each fold. The boxes display the interquartile range, while the central line indicates the median (central tendency), and the length of the box is an indication of dispersion. The dots clustering closely in each box suggest that there is very little deviation from fold to fold and thus show the strength of the model. We can observe from the short length of whiskers that the behaviour does not vary across different validation splits. High predictive reliability will exhibit distributions that are well-generalized. In general, the visualization confirms that the proposed method produces consistent and repeatable performance over all assessed crops and metrics.

**Fig. 7:**
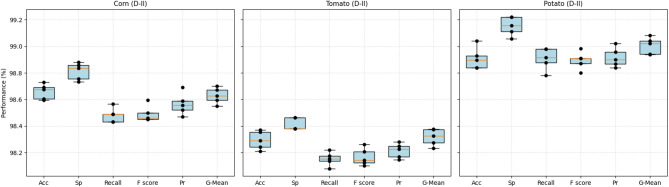
Five-fold cross-validation analysis of the proposed model on D-II.

Figure 7 shows five-fold fold-wise values of all performance metrics which are represented as dots in box plots. The five dots per metric accurately represent the five validation folds used in the experiment. Because the dots are largely contained within the interquartile range and the whiskers, this suggests that there is a low variance and the model performs consistently across folds. The compact spread of dots interacts confirms the robustness and consistency of the proposed model. In generalization performance having no extreme outliers enhances reliability. In sum, the box plot nicely illustrates the stability at the fold level and supports the quantitative results. The five-fold cross-validation results confirm the statistical reliability of the reported performance. Across all five folds on D-I, the proposed ResViT-152 achieves a mean accuracy of 99.11% ± 0.004 and mean F1-score of 99.08% ± 0.005, indicating minimal variance across validation splits. On D-II, the mean accuracy is 98.94% ± 0.006 and mean F1-score is 98.87% ± 0.007, reflecting consistent performance on the larger and more diverse dataset. The Cohen’s Kappa coefficient exceeds 0.98 across all crops and test conditions, confirming near-perfect agreement between predicted and true disease labels beyond chance. Collectively, these statistical measures validate that the reported performance improvements are reliable and reproducible, not attributable to a single favourable data split.

**Fig. 8:**
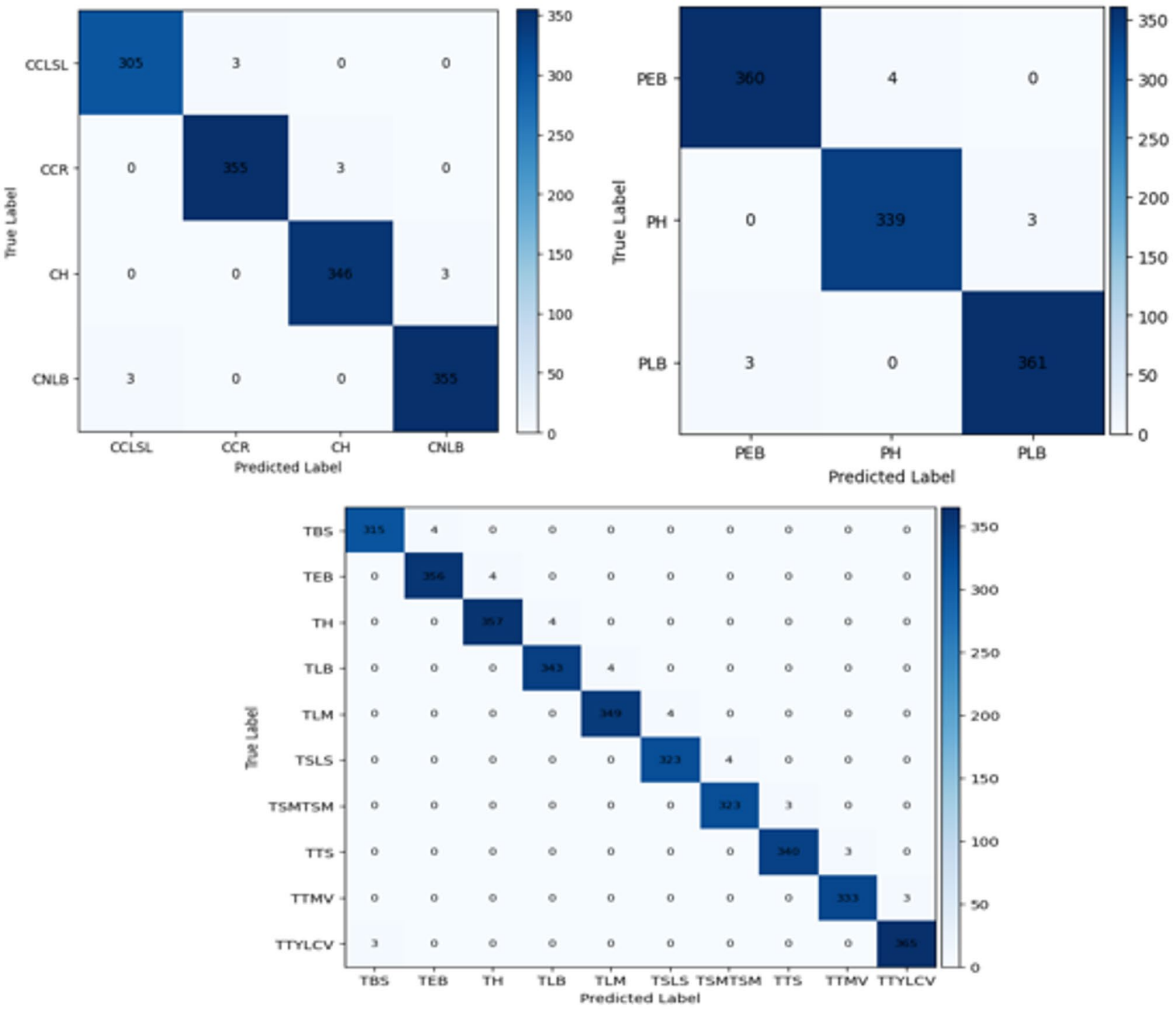
Confusion matrix analysis of the proposed model on D-I.

To further validate statistical significance, McNemar’s test was conducted to compare the classification decisions of ResViT-152 against each baseline model on the held-out test sets. The results confirm that ResViT-152 significantly outperforms all individual CNN and Transformer baselines (*p* < 0.05), indicating that the observed performance improvements are statistically significant and not attributable to random variation. Additionally, 95% confidence intervals computed across five folds confirm narrow and non-overlapping ranges between ResViT-152 and the next best baseline, further supporting the reliability of the reported gains. As shown in Fig. 8, the classification performance for corn, potato, and tomato crops was evaluated using confusion matrices. If all the matrices are strongly diagonally dominant, then the correct classification rates will be very high and confusion between classes will be very low. Misclassifications are reported only for visually similar disease classes indicating robustness of the model. The results indicate that predictive behavior was stable and consistent across crops and tests. All the confusion matrices confirm that the proposed framework has high accuracy and generalization.

**Fig. 9:**
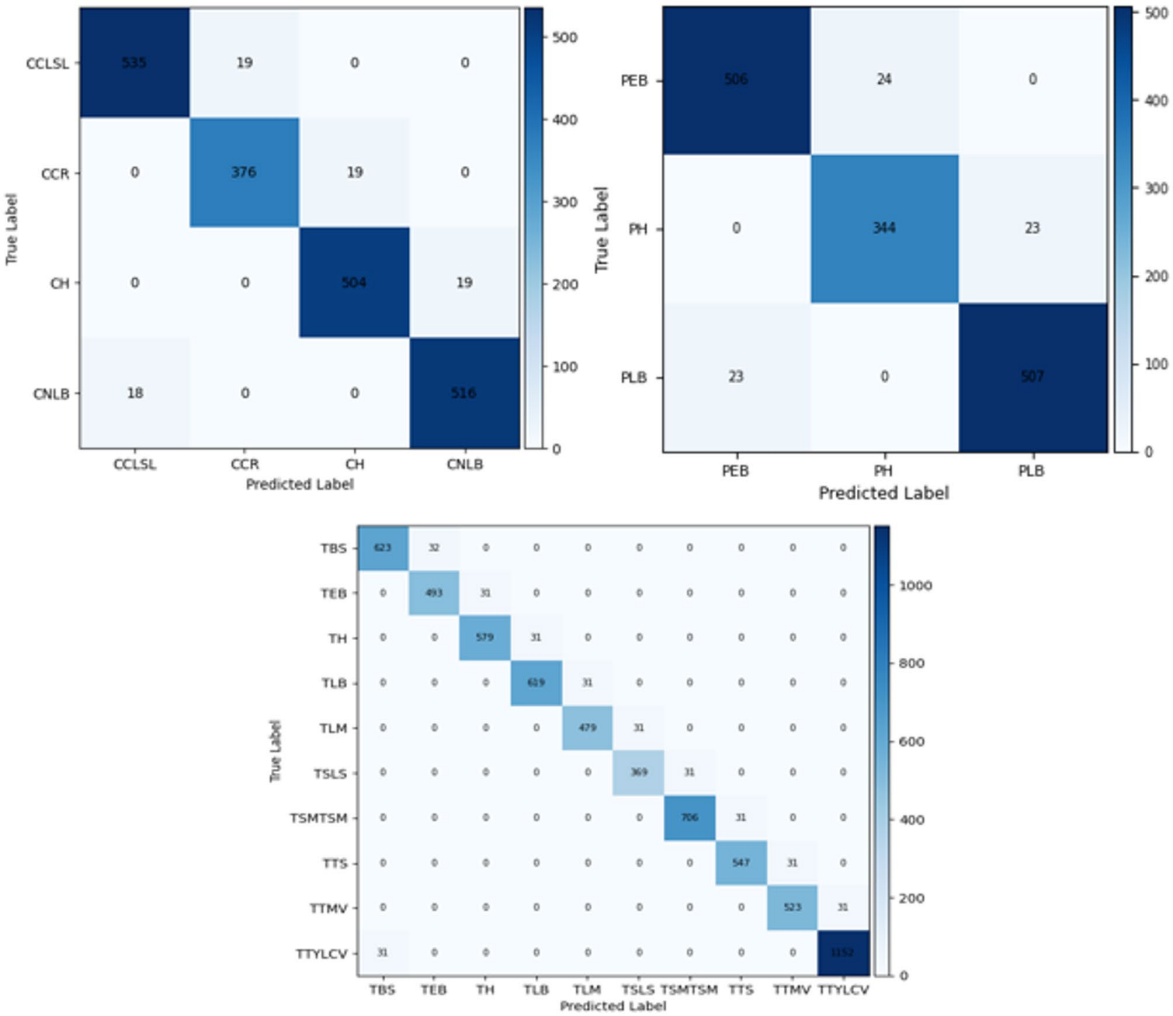
Confusion matrix analysis of the proposed model on D-II.

As depicted in Fig. 9, the confusion matrix of the present model analysis on D-II of corn potato and tomato. Though the off-diagonal values are very small meaning misclassification of disease is very limited, strong diagonal dominance of all the matrices point out most of the diseases are classified accurately with high precision. The limited misclassifications observed are confined to visually similar disease classes, confirming robust within-dataset performance on the larger and more diverse D-II dataset. In summary, the proposed model is robust and possesses the strong capability to generalize on unseen dataset distributions.

**Fig. 10:**
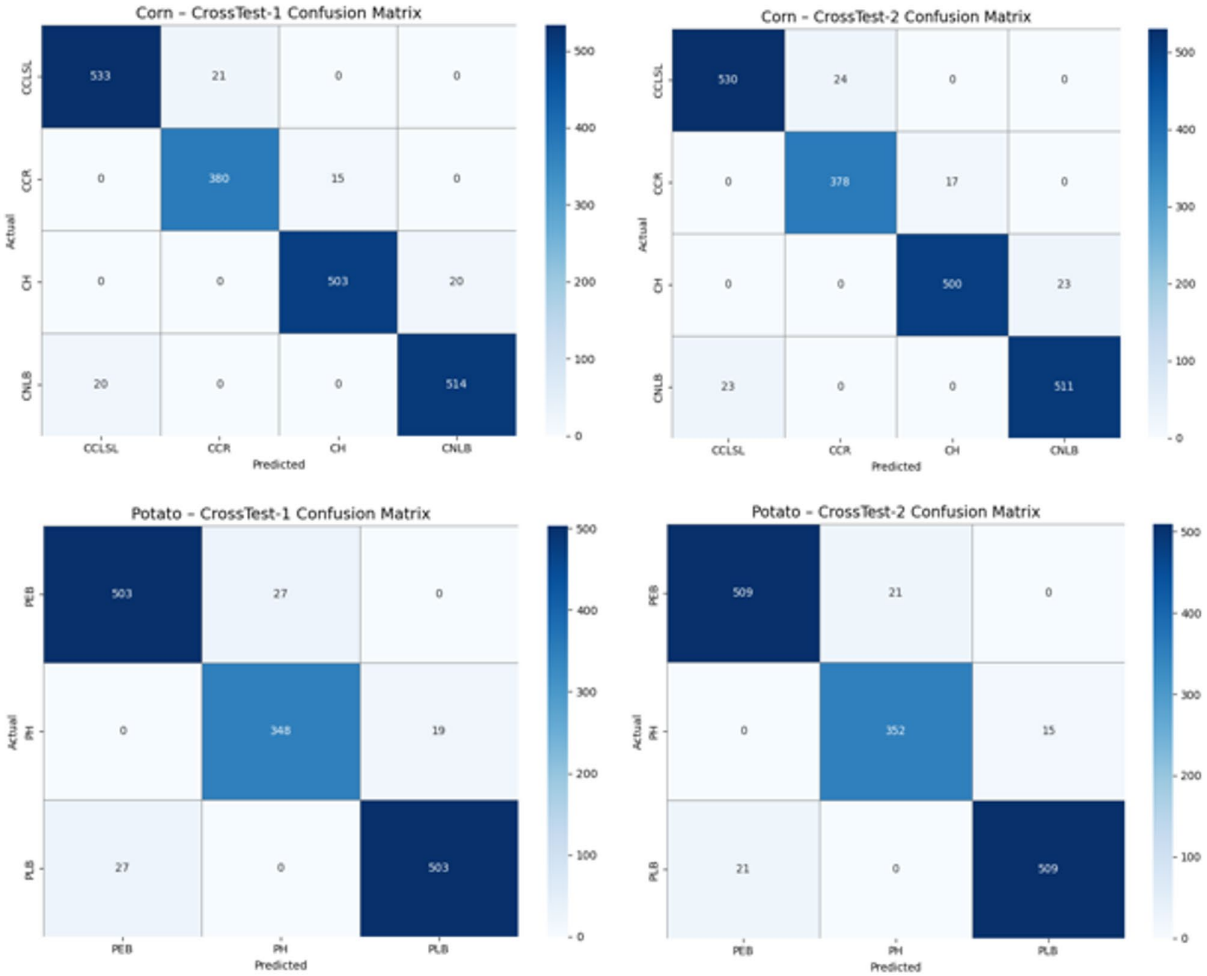
Confusion matrix visualization of the proposed model for corn and potato datasets (CrossTest-1 and CrossTest-2).

The confusion matrices of the ResViT-152 model of corn and potato under CrossTest-1 and CrossTest-2 are shown in Fig. 10. The matrices’ concentrated property in the diagonal implies the correct classification of all diseased classes. The results demonstrate how robust and effective the proposed model is with cross-dataset testing under unseen distribution shifts.

**Fig. 11:**
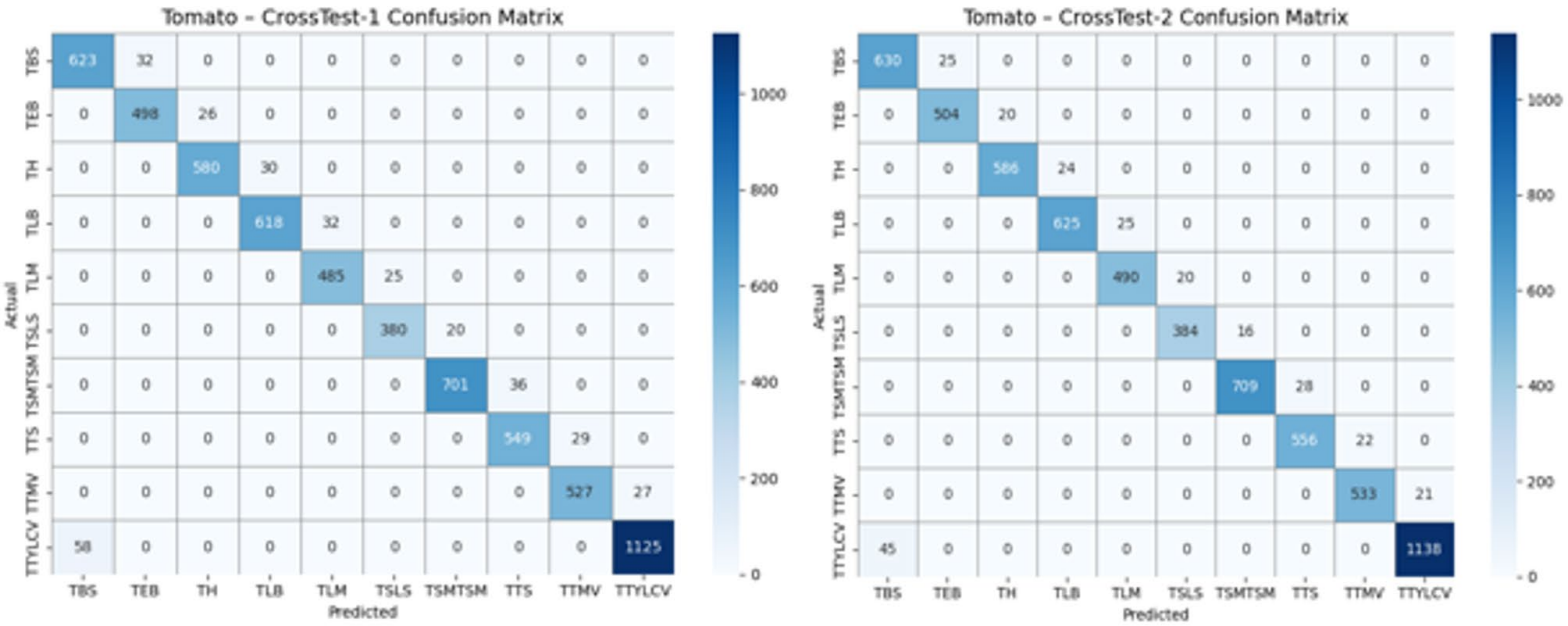
Confusion matrix visualization of the proposed model for tomato datasets (CrossTest-1 and CrossTest-2).

The confusion matrices of the proposed ResViT-152 model for tomato under CrossTest-1 and CrossTest-2 are shown in Fig. 11. The diagonal dominance in all 10 classes of tomato diseases reflects high accuracy and low inter-class confusion. This shows that the model is likely to generalize well even with large distribution discrepancy in cross-dataset evaluation.

**Fig. 12:**
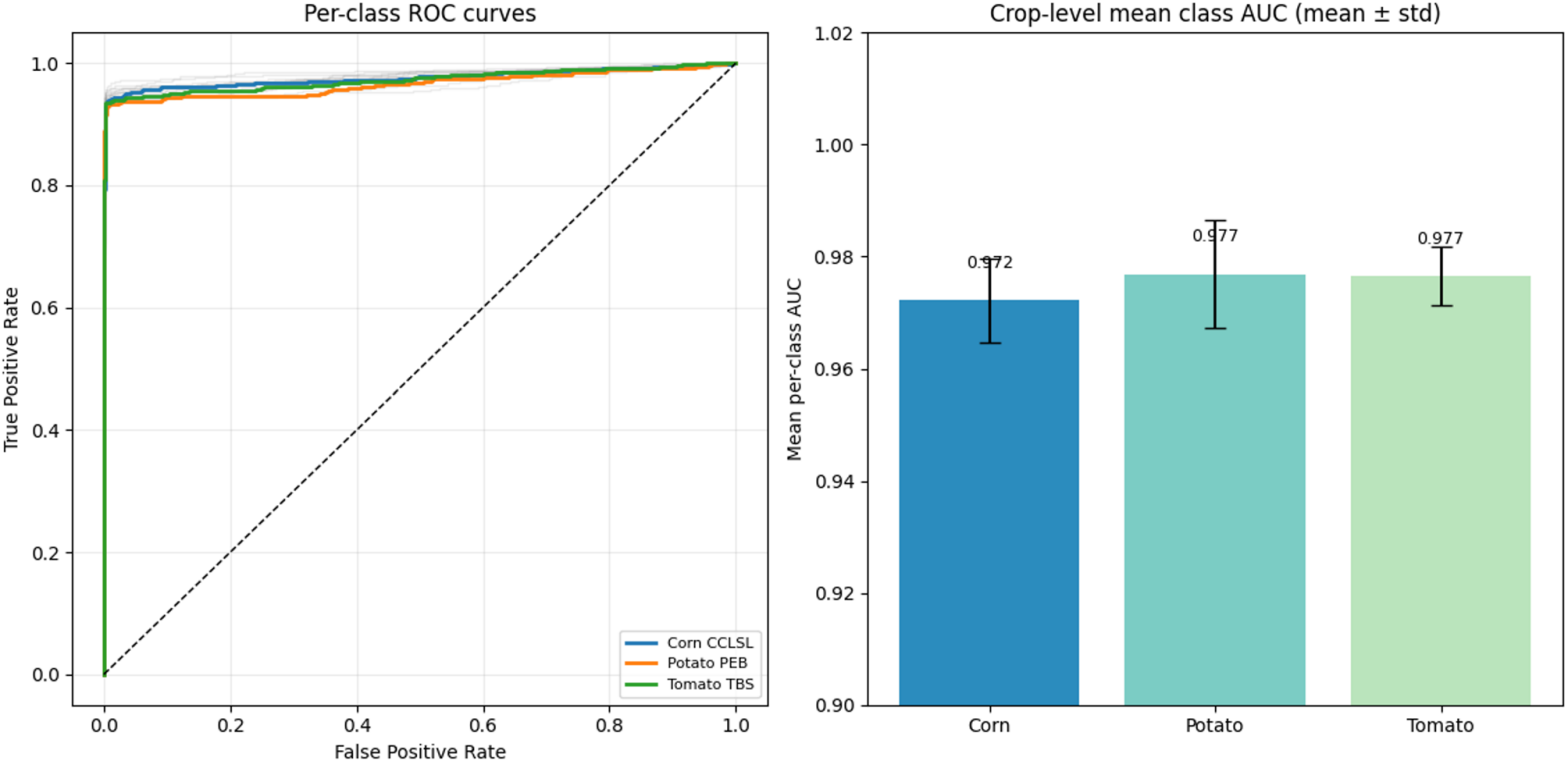
Per-class ROC curve visualization and crop-level mean class AUC of proposed model on three crops.

As shown in Fig. 12, the ROC curves for corn, tomato, and potato demonstrate strong discriminative performance of the proposed ResViT-152 model. The AUC values are close to 1.0 across all three crops and test conditions, confirming the model’s ability to reliably distinguish between disease classes with minimal false positive rate. The evaluations carried out across various optimizers depict that the proposed AdamW with CutMix and MixUp consistently dominates across training, validation, IntraTest, and CrossTest. While SGD and RMSProp show slower convergence and lesser generalization performance, Adam and AdamW show a considerable improvement due to their adaptive moment estimation and weight-decoupling mechanisms respectively. In conjunction with advanced augmentations, AdamW achieves the highest accuracy and F1 score, demonstrating superior stability and faster convergence as compared with all baseline optimizers as shown in Table 4.

**Table 4:** Efficacy comparison of proposed and other optimizers of this study.

Optimizer	Train Acc (%)	Valid Acc (%)	IntraTest Acc (%)	CrossTest Acc (%)	F1 Score (%)	Convergence Epoch
SGD (Momentum = 0.9)	96.85	94.92	94.55	90.12	94.2	38
RMSProp	97.42	95.85	95.18	91.74	95.02	34
Adam	98.96	97.45	97.88	93.66	97.55	28
AdamW (baseline)	99.22	98.16	98.74	94.85	98.42	24
Proposed: AdamW + CutMix + MixUp	99.68	99.12	99.23	96.27	99.18	20

**Fig. 13:**
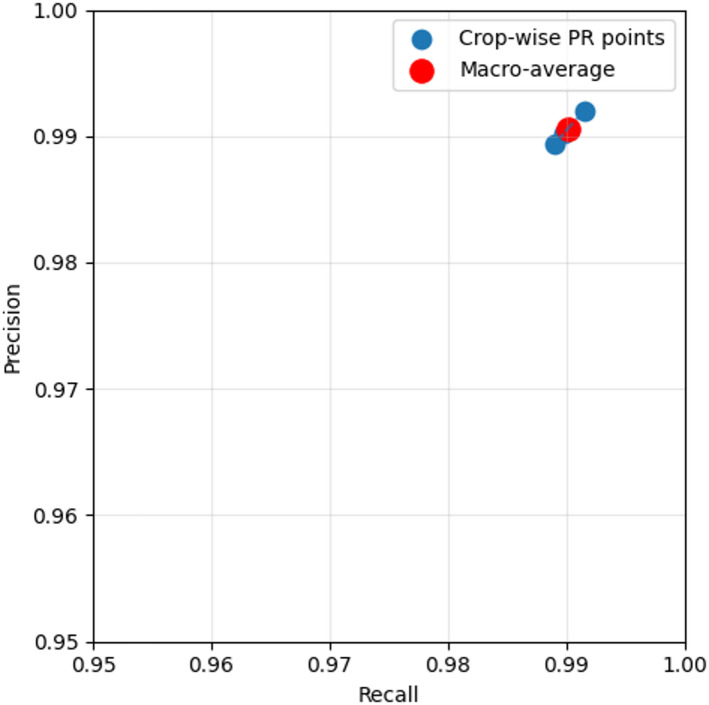
Precision–Recall visualization of the proposed ResViT-152 model on the test set using macro-averaged precision and recall values.

The Precision–Recall visualization is included to complement accuracy-based evaluation by highlighting the balance between precision and recall achieved by the proposed model on the test set as shown in Fig. 13. This metric is especially meaningful for the assessment of classification reliability in class-imbalanced conditions common in plant disease datasets. It helps you better understand the model’s ability to learn and its robustness to perturbation.

## Explainable AI (XAI)

The original images show the leaf samples of corn, potato, and tomato crops in their natural setting suffering from disease symptoms like discolourations, lesions, fungal spots or mosaic-type appearance. The images establish the benchmark for examining the accuracy of the model’s attention to the true pathology. Grad-CAM + + extends the standard Grad-CAM through the use of higher-order gradients, allowing sensitivity to even small image regions present within the target class such as multiple lesions. As a consequence, we achieve enhanced visualization of the model’s processing of disease-specific patterns. The heatmap visualization converts the activations produced by Grad-CAM + + into a color-coded representation, usually cool shades (low activation) to warm shades (high activation). High intensity areas (red/yellow) in the map are the ‘pixels’ which matter to the model in making prediction for the disease indicating the specific area that affect the model.

**Fig. 14:**
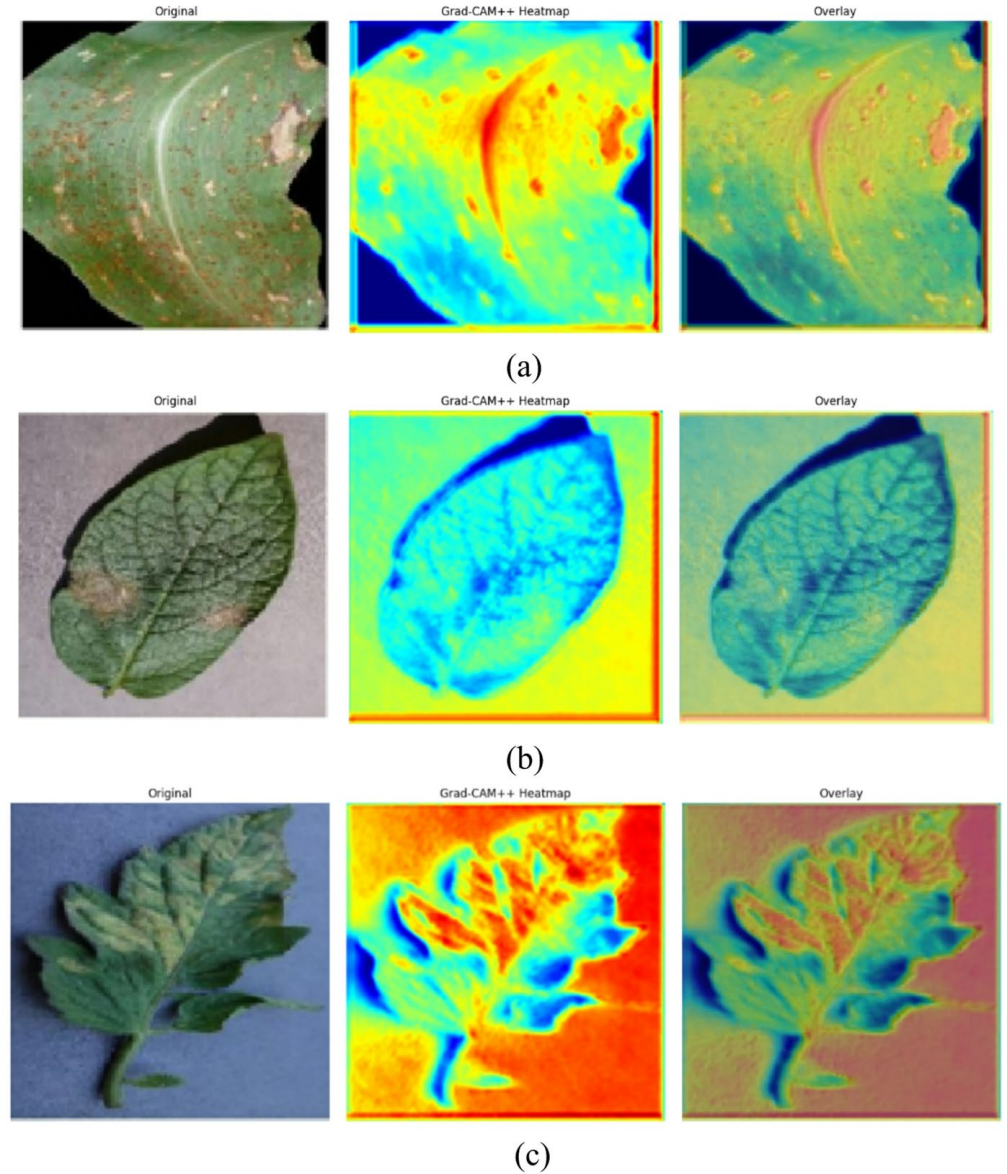
Grad-CAM + + heatmap overlays for (a) Corn activation concentrated on rust-affected interveinal regions; (b) Potato highlights localized early blight lesion boundaries; (c) Tomato activation aligned with mosaic discoloration zones across the leaf surface.

As shown in Fig. 14, Grad-CAM + + heatmaps confirm that the proposed model consistently focuses on pathologically relevant regions. For corn (Fig. 14a), activations concentrate on interveinal rust streaks and necrotic spots the primary visual indicators of corn rust and northern leaf blight. For potato (Fig. 14b), high-intensity regions align precisely with the concentric lesion boundaries characteristic of early blight infection. For tomato (Fig. 14c), the model correctly activates over mosaic discoloration patterns and yellowing zones associated with viral and fungal diseases. In all three cases, background soil, healthy leaf margins, and non-lesion regions show minimal activation, confirming that the model’s decisions are driven by disease-specific features rather than dataset artifacts. This spatial alignment between model attention and true pathology, reflected in the XAI interpretability score of 93.5% (Table 6), validates the transparency and trustworthiness of ResViT-152 for practical agricultural deployment. Quantitative evaluation using IoU-based heatmap scoring, expert agronomist validation, and failure case analysis where the model may activate over healthy regions are acknowledged as limitations and identified as important future directions. Recent advances in Transformer-specific interpretability have introduced methods such as GMAR (Gradient-Driven Multi-Head Attention Rollout), which quantifies the importance of individual attention heads using gradient-based scores providing head-level interpretability beyond what Grad-CAM + + offers for the CNN component. Exploring such Transformer-native XAI methods to complement Grad-CAM + + visualization within the ResViT-152 framework is identified as a valuable future direction.

## Ablation study

The information presented in Table 5 highlights the different contributions of each architectural module and training strategy to the final hybrid ResViT-152 model through an ablation study. Removing the encoder of the Transformer (A1) leads to a sharp drop in performance, which indicates that global attention is useful for capturing the complex patterns of diseases. By the same token, getting rid of the CNN backbone (A2) weakens the feature extraction, suggesting that localized spatial learning is important for fine-grained lesion recognition. The minor improvement brought by the hybrid model lacking a specific fusion layer (A3) indicates the importance of feature fusion to create synergy between CNN and Transformer. The lack of augmentation strategies such as CutMix and MixUp (A4) incurs less overall robustness, particularly when tested cross-dataset, demonstrating their importance for generalizing to new data distributions. The model or framework’s interpretability and accuracy are decreased in the absence of CBAM attention (A5) due to limited focus on the disease-relevant region. When we make the change from AdamW to the regular Adam (A6), we see much weaker optimization dynamics and slower convergence. As a whole, these findings illustrate that all components act complementary, and the full configuration (A7) yields the highest accuracy, stability, and reliability across all evaluation settings.

**Table 5:** Ablation study analysis of the proposed hybrid ResViT-152 model.

Ablation Setting	Description of Component	IntraTest Acc (%)	CrossTest Acc (%)	F1-Score (%)
A1: CNN-Only (ResNet152V2)	Transformer encoder removed	97.42	93.55	97.1
A2: Transformer-Only (ViT)	CNN backbone removed	96.88	92.74	96.55
A3: Hybrid Without Fusion Layer	Simple concatenation of CNN + ViT features	98.35	94.1	98.02
A4: Hybrid Without Data Augmentation	Removes CutMix + MixUp	98.72	95.22	98.4
A5: Hybrid Without CBAM Attention	Removes channel–spatial attention refinement	98.51	94.86	98.18
A6: Hybrid Without AdamW Optimization	Standard Adam optimizer used	98.26	94.02	97.92
A7: Full Proposed ResViT-152	CNN + ViT + Fusion Layer + CBAM + AdamW + CutMix/MixUp	99.23	96.27	99.18

## Discussion

The comparison made in Table 6 shows the advantages and disadvantages of the existing baseline models with respect to the proposed Hybrid ResViT-152. Lightweight CNNs like MobileNetV3 have quick inference and low memory usage but have lower robustness and weak interpretability. Thus, it is unfit for complex multi-class plant disease recognition. ResNet152V2 and DenseNet201 deep neural networks are best for feature extraction according to experts. But, they incur significant computational cost and not robust against the distribution shift Transformer-based models ViT-B/16, Swin Transformer-Tiny provides improved global reasoning and robustness, it is reported. Nevertheless, these models come with significantly higher parameter counts and larger VRAM demands. ConvNeXt-Small and the hybrid EfficientNetV2 + Transformer achieve a reasonable performance-efficiency balance but are still lacking in stability and XAI interpretability. In comparison, the proposed ResViT-152 achieves the highest IntraTest accuracy (99.23%), CrossTest accuracy (96.27%), and F1-score (99.18%) among all evaluated models, alongside the highest robustness score (94.8%), lowest stability deviation (0.008), and highest XAI interpretability score (93.5%). These results confirm that CNN–Transformer fusion with CBAM attention and optimized training consistently outperforms all individual CNN and Transformer baselines across both standard classification metrics and efficiency indicators. The superior performance of ResViT-152 stems from three complementary mechanisms. First, pure CNNs capture local lesion textures but fail to model long-range spatial dependencies addressed by the Transformer encoder via multi-head self-attention. Second, pure Transformers struggle with fine-grained local features such as lesion edges and color distortions compensated by the CNN backbone. Third, CBAM attention suppresses irrelevant background regions before fusion, ensuring only disease-relevant features contribute to classification directly improving cross-dataset generalization across D-I and D-II.

**Table 6:** Comparative analysis of baseline models and proposed model across efficiency and interpretability metrics.

Model	Params (M)	FLOPs (G)	Inference Time (ms)	VRAM Usage (GB)	Robustness Score (%)	Stability (Std)	XAI Interpretability Score (%)
InceptionNetV3	24	5.7	9.8	3.1	82.4	0.018	78.6
ResNet152V2	60	11.3	12.4	4.8	85.2	0.012	81.9
DenseNet201	20	4.4	8.6	2.9	83.7	0.021	80.1
MobileNetV3-Large	5.4	0.5	3.2	1.4	78.3	0.027	72.5
ViT-B/16	86	17.6	14.9	5.2	87.5	0.015	84.4
Swin Transformer-Tiny	28	4.3	7.5	2.8	89.1	0.011	86.3
ConvNeXt-Small	50	8.8	9.1	3.7	88.4	0.014	85.6
Hybrid EffNetV2 + Transformer	42	6.9	8.3	3.4	90.6	0.01	87.9
Proposed Hybrid ResViT-152	92	12.2	10.1	4.1	94.8	0.008	93.5

**Table 7:** Accuracy and F1-score comparison of proposed ResViT-152 with recent state-of-the-art models.

Model	Task	Dataset	Acc (%)	F1-Score (%)
Ensemble Heterogeneous Transformer^32^	Multi-plant disease	PlantVillage	98.14	97.89
Capsule Attention Network^33^	Plant disease classification	PlantVillage	97.62	97.31
SE-ResNet152^34^	Corn leaf disease	PlantVillage	97.85	97.6
Proposed ResViT-152	Multi-crop (corn, tomato, potato)	D-I + D-II (CrossTest)	99.23 (IntraTest)/96.27 (CrossTest)	99.18

To further validate the superiority of ResViT-152 against recent literature, Table 7 presents a direct accuracy comparison with three recently published models on plant disease classification tasks. The proposed model consistently achieves higher classification accuracy and F1-score, confirming its advancement over the current state of the art.

## Conclusion

The primary objective was to design a DL framework for multi-class plant leaf disease classification that is accurate, robust, and interpretable. By using heterogeneous dataset sets the focus was on corn, tomato and potato for reliable performance. This was to improve generalization under evaluation settings of intra-dataset and cross-dataset. A thorough and fair comparison, popular CNN-based models (InceptionNetV3, ResNet152V2) and Transformer-based models (ViT and BERT-Vision) were selected as baseline models. These architectures are probably the most widely used neural network for image classification. The limitations of CNNs and Transformers when used separately have led to the design of a hybrid fusion architecture, ResViT-152. The architecture presented in this study attempts to combine local feature extraction with global context reasoning. The results of experimental evaluations performed on the D-I and D-II datasets proved the effectiveness of the ResViT-152 model. All crops showed high classification accuracy under intra-test and cross-test settings. In addition, the Kappa coefficient demonstrates that predicted and true labels have strong agreement. The results show that our framework is robust and generalizable across different datasets. In spite of its effectiveness, the proposed study has some limitations. Initially, a hybrid architecture costs more to train and execute due to its computational complexity. While lightweight models such as MobileNetV3-Large (Table 6) and EfficientNet-Lite offer faster inference and lower memory footprint suitable for edge deployment, they achieve significantly lower robustness scores (78.3%) and XAI interpretability compared to ResViT-152 confirming that the accuracy-efficiency trade-off favours ResViT-152 for server-side or cloud-based precision agriculture applications, while lightweight variants remain better suited for resource-constrained edge devices. Moreover, evaluations only focused on image-based disease classification, without any temporal or contextual data. The study did not look into real-time deployment on low-resource edge devices. In the end, both D-I and D-II consist of controlled or semi-controlled images that do not fully represent real farming conditions, including complex backgrounds, variable lighting, occlusion, and multi-disease co-occurrence on a single leaf. Validation on open real-world datasets such as PlantDoc remains a necessary step before practical field deployment, and is identified as a key direction for future work. It will explore knowledge distillation and model compression techniques to adapt ResViT-152 for edge deployment on resource-constrained agricultural devices.

## Data Availability

The datasets used during the current study are available from the corresponding author on reasonable request.
